# The Role of Viral Infection and Microbial Dysbiosis in Glaucoma: From Pathogenesis to Therapeutic Strategies

**DOI:** 10.3390/v18030310

**Published:** 2026-03-02

**Authors:** Xiaobo Wang, Ji Zhang, Jiawei Chen, Qiuling Huang, Xuanchu Duan, Wenxiang Zhu

**Affiliations:** 1 Aier Academy of Ophthalmology, Central South University, Changsha 410083, China; wxiaobo@csu.edu.cn (X.W.); jiawei_chen20@foxmail.com (J.C.); 2 Aier Glaucoma Institute, Hunan Engineering Research Center for Glaucoma with Artificial Intelligence in Diagnosis and Application of New Materials, Changsha Glaucoma Diagnosis and Treatment Technology Innovation Center, Changsha Aier Eye Hospital, Changsha 410015, China; 3 Department of Ophthalmology, The Second Xiangya Hospital, Central South University, Changsha 410083, China; 8301190422@csu.edu.cn

**Keywords:** glaucoma, viral infection, gut–eye axis, autophagy, neuroinflammation

## Abstract

Glaucoma is a leading cause of irreversible blindness, yet vision loss often progresses despite effective intraocular pressure (IOP) control, suggesting the involvement of non-hydrodynamic mechanisms. This review explores the potential synergistic interaction between viral persistence and microbial dysbiosis in pathogenesis. While acknowledging that current evidence regarding the microbiome is largely associative and derived from small cohorts or animal models, we analyze how these environmental insults may disrupt autophagic flux and induce immune dysregulation to drive chronic neuroinflammation. Furthermore, we explore theoretical therapeutic strategies targeting this distinct pathological nexus, ranging from metabolic restoration of the gut–eye axis to the repurposing of advanced nanocarriers to overcome ocular barriers. This framework lays the groundwork for next-generation, etiology-based precision management.

## 1. Introduction

Glaucoma is a leading cause of irreversible blindness worldwide. It is characterized by distinct epidemiological variations across geographical regions and ethnicities. Primary open-angle glaucoma (POAG) is the most prevalent subtype, with an increased incidence with age and heterogeneous clinical patterns among different ethnic groups [1]. Normal-tension glaucoma is especially prevalent in Asian populations, accounting for over 90% of open-angle glaucoma cases in Japan, compared to 30% in Europe [2,3]. Such discrepancies suggest that pathogenic drivers extend well beyond the traditional risk factor of elevated intraocular pressure (IOP) [4]. Together with molecular similarities to Alzheimer’s disease, these epidemiological findings support reclassifying glaucoma as a systemic neurodegenerative disorder [1,5,6,7]. Increasing evidence indicates that environmental factors, particularly viral infection and microbial dysbiosis, may influence glaucoma progression through modulation of immune-inflammatory pathways and autophagic homeostasis [8,9,10]. This interaction provokes abnormal protein aggregation by disrupting critical autophagic pathways, such as the VPS39/STX17 complex [11]. Concurrently, microbiome research has unveiled the gut–eye axis, indicating that dysbiosis in intestinal, oral, and ocular surface microbiota may trigger systemic or localized innate immune responses via the lipopolysaccharides (LPS)-TLR4 pathway, culminating in chronic neuroinflammation and subsequent optic nerve damage [12,13].

While the independent roles of viral infection and microbial dysbiosis in glaucoma have been reported, a comprehensive synthesis of their potential interaction with host autophagy and immune regulation is currently lacking. This etiopathogenesis involving multiple distinct insults may explain why optic nerve function continues to deteriorate in some patients despite well-controlled IOP. The potential synergy between *Helicobacter pylori* (*H. pylori*) and viral infections, alongside the role of microbial metabolites in neuroprotection or injury, offers new perspectives on glaucoma heterogeneity [14,15,16]. However, most available evidence is derived from animal models or cross-sectional human studies, and clinical translation remains limited by interspecies differences, cohort heterogeneity, and confounding factors.

This review systematically analyzes the potential synergistic interactions between viral infection and microbial dysbiosis in the pathogenesis of glaucoma. We focus particularly on how these factors converge to disrupt immune regulation and autophagic flux. Based on these insights, we propose a “host-virus-microbiome” framework. However, it is important to note that this model remains largely conceptual. While direct causal links have been established for specific viral infections such as Zika virus (ZIKV) and herpes simplex virus (HSV) in animal models, evidence connecting the human microbiome to glaucomatous neurodegeneration is currently predominantly associative. Accordingly, we explicitly distinguish between mechanistically proven pathways and hypothetical interactions throughout the text. We conclude by evaluating etiology-based therapeutic strategies, including metabolic restoration along the gut–eye axis and advanced nanotechnology delivery systems, which are designed to overcome ocular barriers and establish next-generation precision management.

## 2. Viral-Mediated Pathogenic Mechanisms in Glaucoma

**Figure 1:**
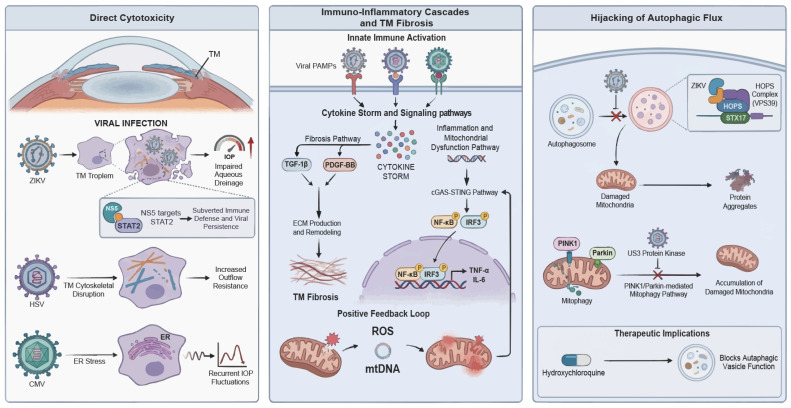
Integrated molecular landscape of viral-induced glaucoma. Schematic representation of the hypothesized mechanisms by which viral infection disrupts ocular homeostasis through three convergent pathogenic pathways. Direct cytotoxicity and tissue tropism: ZIKV displays marked tropism for TM cells; NS5-mediated STAT2 degradation compromises host immunity and impairs aqueous drainage. HSV infection disrupts cytoskeletal integrity, thereby increasing outflow resistance, whereas CMV-induced ER stress drives recurrent IOP fluctuations. Immuno-inflammatory cascades and fibrosis: Viral PAMPs activate innate immune sensors (e.g., TLRs/RIG-I) to initiate a cytokine storm. Subsequent activation of the cGAS-STING axis triggers NF-κB and IRF3 translocation, releasing pro-inflammatory cytokines, such as IL-6 and TNF-α. This signaling upregulates TGF-β1 and PDGF-BB, driving excessive ECM deposition and TM fibrosis. Autophagic hijacking and mitochondrial dysfunction: ZIKV blocks autophagosome-lysosome fusion by targeting the VPS39-STX17 complex. Conversely, HSV inhibits PINK1-Parkin-mediated mitophagy. These defects result in ROS accumulation and mtDNA release, fueling a positive feedback loop of neuroinflammation and cell death. ZIKV, Zika virus; TM, trabecular meshwork; NS5, non-structural protein 5; STAT2, signal transducer and activator of transcription 2; HSV, herpes simplex virus; CMV, cytomegalovirus; ER, endoplasmic reticulum; IOP, intraocular pressure; PAMPs, pathogen-associated molecular patterns; TLRs, Toll-like receptors; RIG-I, retinoic acid-inducible gene I; cGAS-STING, cyclic GMP-AMP synthase-stimulator of interferon genes; NF-κB, nuclear factor kappa-light-chain-enhancer of activated B cells; IL-6, interleukin-6; TNF-α, tumor necrosis factor-alpha; IRF3, interferon regulatory factor 3; TGF-β1, transforming growth factor-beta 1; PDGF-BB, platelet-derived growth factor-BB; ECM, extracellular matrix; VPS39, VPS39 subunit of HOPS complex; STX17, syntaxin 17; PINK1, PTEN induced kinase 1; ROS, reactive oxygen species; mtDNA, mitochondrial DNA.

Viral infection serves as a critical environmental trigger capable of disrupting the delicate equilibrium between ocular immune privilege and aqueous humor dynamics. Mounting evidence suggests that viral pathogens do not merely invade key ocular tissues but actively hijack host molecular machinery to initiate deleterious cascades. It should be noted, however, that most mechanistic insights are derived from experimental animal models or in vitro systems, and their relevance to human glaucoma remains to be fully established. This section details the pathological network of viral involvement in glaucoma, focusing on three key mechanisms: direct cytotoxicity, immuno-inflammatory remodeling, and autophagic dysregulation (Figure 1).

### 2.1. Viral Tissue Tropism and Direct Cytotoxicity

This section focuses on ZIKV, HSV, and cytomegalovirus (CMV), which currently provide the strongest experimental evidence linking viral infection to trabecular meshwork (TM) injury or defined glaucoma phenotypes. Other ocular viruses, such as varicella-zoster virus (VZV) and human immunodeficiency virus (HIV), are acknowledged but are not discussed in detail because glaucoma-specific mechanistic data remain limited.

The direct infection of aqueous outflow pathways and optic nerve tissues constitutes the initiating event in IOP elevation and neurodegeneration. Research confirms that ZIKV, HSV, CMV possess distinct invasive capacities for specific ocular structures. ZIKV demonstrates a specific tropism for the TM in *Ifnar1*^−/−^ mouse models, where infection directly leads to impaired aqueous drainage and subsequent ocular hypertension [9]. In parallel, studies have highlighted the susceptibility of human TM cells to CMV infection, which may exacerbate glaucomatous pathology through distinct inflammatory mechanisms [8]. Such variations in viral tropism dictate the diversity of clinical manifestations: ZIKV invades the host by binding to specific receptors on neural progenitor cells and retinal pigment epithelium (RPE), exhibiting high infectivity and pro-apoptotic effects in fetal RPE cells, while showing lower infection rates in retinal stem cells [17,18,19,20]. Nevertheless, whether these tissue-specific infection patterns directly translate into chronic glaucomatous neurodegeneration in humans remains uncertain.

In contrast, HSV and CMV are primarily characterized by persistent damage resulting from chronic or latent infections. Mechanistically, HSV infection has been shown to directly compromise TM cell cytoskeletal architecture, thereby increasing outflow resistance [21]. Patients with neonatal HSV keratitis may develop refractory bilateral glaucoma even nine years post-infection [22]. CMV leverages its latency to cause recurrent IOP fluctuations [23,24], a phenomenon potentially linked to virus-induced endoplasmic reticulum stress. Previous studies indicate that reducing endoplasmic reticulum stress via chemical chaperones can prevent glaucomatous phenotypes. We therefore hypothesize that viral persistence may chronically activate these stress pathways, leading to intracellular metabolic dysfunction and pro-inflammatory cytokine release [25]. This hypothesis requires confirmation in longitudinal human studies. Viral components such as ZIKV non-structural protein 5 can subvert host immune defenses by targeting STAT2 for degradation, fostering intraocular viral persistence and exacerbating tissue damage [26,27].

### 2.2. Immuno-Inflammatory Cascades and TM Fibrosis

The immuno-inflammatory response represents the core pathological axis of viral glaucoma [28]. Upon entry, viral pathogens activate the innate immune system via pattern recognition receptors, particularly Toll-like receptors and RIG-I-like receptors, precipitating a cytokine storm that fundamentally alters the intraocular microenvironment. Following HSV-1 infection of TM cells, the transforming growth factor-β1 and platelet-derived growth factor-BB signaling pathways are markedly up-regulated, promoting the expression of profibrotic factors and the secretion of monocyte chemoattractant protein-1 [21]. This sustained inflammatory milieu disrupts the balance between matrix metalloproteinases and their inhibitor, leading to excessive extracellular matrix deposition and remodeling, ultimately resulting in TM fibrosis and outflow obstruction.

Intracellular immunoregulatory pathways play a decisive role in this process. TNF receptor associated factor 3 negatively regulates type I interferon responses during infections caused by DNA viruses, specifically HSV-1 [29]; its dysregulation may synergize with TANK-binding kinase 1 via the non-canonical NF-κB pathway to amplify pro-inflammatory cytokine production [30]. Crucially, the activation of the cGAS-STING pathway, which functions as a sensor for viral dsDNA, triggers the nuclear translocation of IRF3 and NF-κB, inducing the release of IL-6 and TNF-α [31,32]. Virus-induced mitochondrial dysfunction can release reactive oxygen species and mtDNA, which in turn reactivates the cGAS-STING pathway. This creates a positive feedback loop involving inflammation and mitochondrial damage, closely recapitulating the neuroinflammatory mechanisms observed in neurodegenerative diseases like AD [33]. These parallels suggest shared inflammatory architectures rather than definitive proof of a unified causal mechanism in glaucoma.

### 2.3. Hijacking of Autophagic Flux and Molecular Regulatory Networks

Beyond direct injury and inflammation, the sophisticated manipulation of host autophagic pathways by viruses has emerged as a significant pathogenic mechanism. ZIKV is currently the only known flavivirus identified as a causative agent of congenital glaucoma, primarily through the specific blockade of autophagic flux. Ahmad et al. revealed that ZIKV inhibits autophagosome-lysosome fusion by modulating the HOPS complex member VPS39 and the SNARE protein STX17 [11]. This blockade results in the accumulation of aberrant protein aggregates and damaged mitochondria due to impaired mitophagy. These factors drive a pathogenic cascade involving uncontrolled ROS production, mitochondrial DNA mutations, and bioenergetic failure. Ultimately, this process may culminate in the death of TM cells.

Interestingly, ZIKV blocks late-stage autophagic flux by inhibiting autophagosome-lysosome fusion, thereby hijacking these vesicles to facilitate viral replication and egress [34]. In contrast, HSV-1 actively subverts mitochondrial quality control by employing its US3 protein kinase to inhibit the PINK1/Parkin-mediated mitophagy pathway. This blockade not only facilitates viral propagation but also exacerbates microglial inflammation and impairs the clearance of protein aggregates, such as amyloid-β [35]. These mechanistic divergences highlight that autophagy modulation is virus-specific rather than uniform, underscoring the need for pathway-selective therapeutic approaches. For instance, hydroxychloroquine, an autophagy inhibitor, has been shown to restrict ZIKV replication in primary human TM cells by blocking the viral exploitation of autophagic vesicles for egress, yet its clinical utility is limited by potential risks of retinal toxicity [11]. Thus, while autophagy represents a promising therapeutic target, current evidence remains preclinical, and broad-spectrum autophagy modulation may pose safety concerns. Precisely targeting the VPS39/STX17 complex or virus-specific regulatory nodes may offer superior clinical promise compared with non-selective autophagy inhibitors.

## 3. The Potential Link Between the Microbiome and Glaucoma

**Figure 2:**
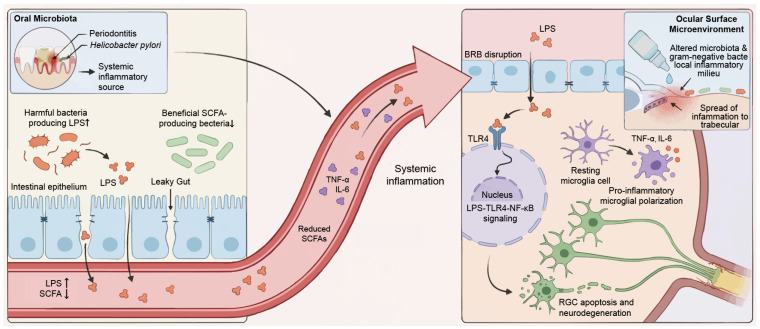
Microbial dysbiosis and the gut–eye axis in glaucomatous neurodegeneration. The schematic illustrates the hypothesized pathological link between peripheral dysbiosis and central ocular injury. Systemic dysbiosis and inflammatory origins: Oral (e.g., periodontitis, *H. pylori*) and intestinal dysbiosis act as primary inflammatory reservoirs. In the gut, pathogenic LPS-producing bacteria expand while beneficial SCFA-producing commensals decline. This imbalance compromises epithelial integrity (“leaky gut”). Translocation and BRB disruption: Bacterial endotoxins (e.g., LPS) and cytokines breach the intestinal barrier to enter systemic circulation. These mediators subsequently traverse the BRB. TLR4-mediated microglial activation: Infiltrating LPS engages TLR4 receptors on retinal microglia. This interaction triggers the NF-κB signaling cascade. Consequently, microglia polarize to a pro-inflammatory state and release neurotoxic cytokines (e.g., TNF-α, IL-6), RGC apoptosis. Ocular surface microenvironment: Gram-negative bacterial overgrowth characterizes local dysbiosis. This shift creates a pro-inflammatory milieu that spreads to the TM. *H. pylori*, *Helicobacter pylori*; LPS, lipopolysaccharides; SCFA, short-chain fatty acids; BRB, blood-retinal barrier; TLR4, Toll-like receptor 4; NF-κB, nuclear factor kappa-light-chain-enhancer of activated B cells; TNF-α, tumor necrosis factor alpha; IL-6, interleukin-6; RGC, retinal ganglion cell; TM, trabecular meshwork.

While viral infection serves as an immediate precipitating factor for glaucomatous onset, microbial dysbiosis constitutes its persistent underlying context. Modern multi-omics research reveals that the microbiome is no longer confined to local commensalism; instead, it deeply regulates retinal ganglion cell (RGC) survival via the gut–eye axis, defined as a long-range communication system utilizing metabolites, immunological signals, and neurotransmitter networks. It should be emphasized that most current evidence supporting microbiome involvement in glaucoma is associative and derived from small human cohorts or experimental models. This section dissects the multifaceted roles of the microbiome in glaucoma through three dimensions: systemic inflammatory drivers, modulation of metabolites and the local microenvironment, and the synergistic pathogenic mechanisms between bacteria and viruses (Figure 2).

### 3.1. Gut–Eye Axis Activation and Systemic Neuroinflammation

Systemic inflammation, propagated by intestinal dysbiosis, acts as the primary pathogenic conduit bridging gut alterations to optic nerve injury. Converging evidence indicates that compromised intestinal barrier integrity often manifests as increased intestinal permeability. This defect facilitates the translocation of bacterial LPS into the systemic circulation. Consequently, such translocation induces a state of chronic low-grade endotoxemia [36,37]. Crucially, recent findings demonstrate that this circulating LPS can traverse the blood-retinal barrier (BRB) to specifically engage TLR4 within the retinal parenchyma and optic nerve. This ligand-receptor interaction triggers the canonical NF-κB signaling cascade, establishing a pro-inflammatory milieu that drives neurodegeneration [38]. This process facilitates the polarization of microglia toward a pro-inflammatory phenotype, releasing cytokines such as TNF-α and IL-6 while activating the complement system, ultimately leading to RGC apoptosis and axonal degeneration [39].

Animal models have demonstrated that TLR4 antagonists, exemplified by naloxone, can intercept this pathological cascade and partially mitigate neural damage, providing robust evidence for the hypothesis that microbial-derived LPS drives glaucoma progression via the TLR4 pathway [13]. Furthermore, in obesity-associated glaucoma models, systemic inflammation induced by gut microbiota disturbances has been shown to infiltrate the intraocular environment through systemic circulation, exacerbating optic nerve inflammation [40].

However, caution is warranted when extrapolating these findings to human glaucoma. Most clinical studies linking gut dysbiosis to glaucoma are cross-sectional, limited by small sample sizes, and subject to significant confounding factors including diet, ethnicity, age, and systemic medications. Thus, it remains difficult to determine whether dysbiosis represents a driver of glaucomatous pathology or a secondary consequence of chronic disease. Therefore, the LPS-TLR4 mechanisms described above are primarily derived from animal models and require further validation in longitudinal human cohorts.

### 3.2. Modulation of Local Microenvironments and Metabolites

Beyond systemic influences, the local microbial microenvironments of the ocular surface and oral cavity are closely linked to glaucoma. Emerging clinical and epidemiological evidence strongly implicates oral dysbiosis in the pathogenesis of glaucoma. Quantitative analyzes reveal that glaucoma patients harbor a significantly higher oral bacterial load compared to healthy controls, suggesting the oral cavity serves as a persistent antigenic reservoir [13]. This microbiological finding is corroborated by large-scale cohort studies and meta-analyzes, which demonstrate a robust association between chronic periodontal disease, tooth loss, and an increased risk of POAG [41,42,43,44]. Furthermore, specific pathogens such as *H. pylori*, often colonizing the oral-gastric axis, have been linked to glaucoma severity [45]. Collectively, these data suggest that oral chronic infection triggers a systemic low-grade inflammatory response, thereby exacerbating neuroinflammation and optic nerve degeneration. On the ocular surface, the chronic administration of preservative-containing anti-glaucoma medications, particularly those containing benzalkonium chloride, imposes a potent environmental selective pressure that fundamentally reshapes the conjunctival microbiome [46]. This chemical stress drives a significant dysbiotic shift characterized by the depletion of protective commensal Gram-positive residents, such as Coagulase-negative Staphylococci, and the predominant overgrowth of opportunistic Gram-negative bacteria [47,48]. Recent high-resolution analyzes further indicate that this bacteriome alteration facilitates the expansion of drug-resistant Gram-negative strains, thereby potentially compromising the outcomes of filtration surgeries [49,50]. This localized dysbiosis triggers a significant remodeling of the tear proteome characterized by an enrichment of immune-related proteins. These proteins comprise factors like complement components and immunoglobulins. Such proteomic alterations not only sustain a pro-inflammatory microenvironment on the ocular surface but may also activate downstream TLR signaling pathways, thereby propagating inflammation to the TM and potentially compromising aqueous humor outflow [51].

Concurrently, the loss of protective microbial metabolites represents another critical mechanism. Under homeostatic conditions, intestinal commensals metabolize dietary substrates to produce essential neuroprotective agents, most notably short-chain fatty acids (SCFAs) and tryptophan derivatives, particularly indolelactic acid and indolepropionic acid [52,53]. Mechanistically, SCFAs, particularly butyrate, regulate microglial function by acting as broad-spectrum inhibitors of histone deacetylases [54,55]. This epigenetic regulation is pivotal for preventing microglial overactivation and suppressing aberrant synaptic pruning; thus, the depletion of these metabolites during dysbiosis leaves RGC vulnerable to neurodegenerative stress [56]. Dysbiosis-induced depletion of these protective metabolites fundamentally compromises the structural integrity of the BRB. Under normal physiological conditions, SCFAs are essential for upregulating the expression of tight junction proteins, including zonula occludens-1 and occludin. These proteins maintain barrier impermeability [57]. However, their reduction leads to a pathological increase in BRB permeability, often described as a phenotype characterized by compromised ocular barrier integrity [58]. This barrier breakdown removes the immune privilege of the eye, rendering the optic nerve increasingly vulnerable to the unchecked infiltration of systemic inflammatory insults and circulating neurotoxins [59].

### 3.3. Systemic Dysbiosis Primes Ocular Viral Susceptibility

We propose that gut dysbiosis may heighten ocular viral susceptibility by disrupting the basal signaling of systemic type I interferons. Previous reviews highlight that type I interferons, specifically IFN-α and IFN-β, represent major effector cytokines in host immunity and can traverse from the periphery to the central nervous system to modulate the functions of microglia, astrocytes, and neurons. This implies that the systemic immune state can directly modulate the local microenvironment of the eye and central nervous system [60].

This implies that systemic immune status may modulate the intraocular microenvironment rather than acting as an isolated peripheral phenomenon. Experimental evidence supports this concept. Hendricks et al. revealed that elevated serum titers of endogenous IFN-α, or systemic IFN-α treatment, markedly restricted HSV-1 corneal invasiveness and prevented the development of stromal disease [61]. This suggests that systemic defense factors are capable of blocking viral spread within ocular tissues.

This systemic support is particularly critical given that locally within the retina, Müller glial cells have been confirmed to be susceptible to viral infections, such as those caused by Dengue virus, and to mount an antiviral response involving inflammatory cytokines and immunomodulatory molecules [62]. Similarly, in the central nervous system, analogous glial cells like astrocytes rely on the transcription factor IRF7 to establish cell-autonomous antiviral responses and limit neurotropic viral replication [63]. We therefore hypothesize that dysbiosis-induced suppression of basal type I interferon signaling may weaken this systemic-local protective axis, rendering Müller cells and TM cells more susceptible to viral reactivation or persistence. However, this proposed link between dysbiosis and ocular viral susceptibility remains speculative and has not yet been directly demonstrated in glaucoma patients. Future studies integrating virome, microbiome, and host immune profiling will be necessary to validate this interaction.

## 4. Therapeutic Strategies and Research Advances

**Figure 3:**
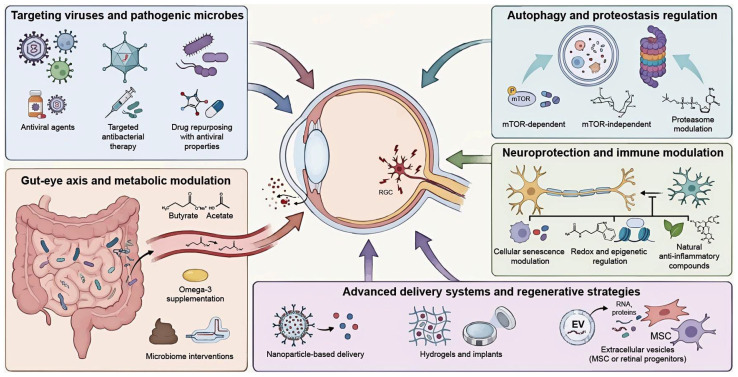
Multidimensional therapeutic framework for virus-associated glaucoma. The schematic illustrates the paradigm shift from IOP-centric management to a holistic strategy. Targeting viruses and pathogenic microbes: Precision antiviral and antibacterial therapies suppress active replication. Drug repurposing leverages secondary antiviral properties of existing ophthalmic medications to mitigate chronic viral persistence. Gut–eye axis and metabolic modulation: Dietary interventions (e.g., Omega-3) and microbial metabolites (e.g., SCFAs: butyrate, acetate) restore intestinal barrier integrity. These modulators attenuate systemic inflammation and dampen ocular immune activation. Autophagy and proteostasis regulation: mTOR-dependent and mTOR-independent pathways enhance lysosomal clearance of protein aggregates. Proteasome modulation supports neuronal protein turnover to alleviate virus-induced proteostatic stress. Neuroprotection and advanced delivery: Targeting cellular senescence and redox pathways (NRF2/Sirt1) counteracts chronic inflammatory stress. Nanoparticle-based systems, hydrogels, and mesenchymal stem cell-derived extracellular vesicles bypass ocular barriers to ensure sustained posterior segment delivery. IOP, intraocular pressure; Omega-3, omega-3 polyunsaturated fatty acids; SCFAs, short-chain fatty acids; mTOR, mechanistic target of rapamycin; NRF2, nuclear factor erythroid 2-related factor 2; Sirt1, sirtuin 1.

Given the complex interplay of viral infection, bacterial co-factors, and systemic dysbiosis, strategies targeting IOP alone are insufficient for comprehensive clinical management. Therapeutic strategies are therefore undergoing a paradigm shift from broad-spectrum antivirals toward a multidimensional framework targeting critical molecular nodes and host microecological resilience. To address the limitations of current pharmacotherapies, we propose a multi-modal therapeutic framework that integrates microbiome-based metabolic restoration, autophagy rescue, and precision nanotherapeutic delivery (Figure 3).

### 4.1. Therapeutic Implications of Virus-Associated Ocular Pathology for Glaucoma

Clinical observations from virus-associated ocular diseases provide important insights into therapeutic strategies relevant to glaucoma pathogenesis. Timely antiviral therapy effectively suppresses viral replication and attenuates intraocular inflammation. For instance, Singh et al. reported clinical resolution using high-dose systemic and topical acyclovir, alongside brimonidine and timolol for IOP control [64]. Although not designed to treat glaucoma directly, this case highlights how virus-driven inflammation and secondary IOP elevation may be pharmacologically mitigated to reduce optic nerve stress.

By contrast, antiviral efficacy appears limited once chronic viral persistence and immune dysregulation are established. Domínguez García et al. reported prolonged corneal viral replication and trabeculitis in Mpox keratitis despite aggressive antiviral therapy, suggesting that viral suppression alone is insufficient to reverse established alterations in aqueous outflow pathways [65]. Similarly, Van Haecke et al. identified *Spiroplasma ixodetis* in a newborn with congenital glaucoma, emphasizing that targeted antimicrobial therapy is essential before structural intervention [66]. Immune-mediated pathology further complicates outcomes. For instance, in hemorrhagic occlusive retinal vasculitis, aggressive intervention with corticosteroids and early vitrectomy failed to prevent severe visual loss [67]. In Behçet uveitis, long-term immunosuppression via fluocinolone acetonide implantation has been linked to CMV endothelitis and secondary glaucoma [68]. These findings highlight the importance of balancing antiviral control with immune modulation.

Drug delivery barriers remain a challenge. Lipid-based nanoparticle systems enhance corneal penetration of antiviral agents [69], while drug repurposing studies indicate that commonly used ophthalmic medications may exhibit secondary antiviral properties that subtly influence viral persistence. Collectively, these findings are most relevant to glaucoma phenotypes characterized by chronic immune activation and suspected infectious or dysbiotic triggers.

### 4.2. Metabolic Modulation and Microbial Restoration of the Gut–Eye Axis for Glaucoma

Targeting the gut–eye axis represents a promising therapeutic avenue for glaucoma, particularly in the context of microbial dysbiosis and viral susceptibility. A large-scale cohort study by Vergroesen et al. demonstrated depletion of butyrate-producing taxa in glaucoma patients, correlating with elevated IOP and increased cup-to-disc ratios [70]. Restoration of microbial-derived metabolites may therefore influence both ocular hydrodynamics and antiviral immune tone. This association remains indirect, and the causal relationship requires further exploration.

SCFAs exert direct metabolic and immunological effects. Butyrate administration lowered IOP in normotensive rat models [71], while phenylbutyrate and sodium acetate suppressed ZIKV replication in human TM cells via FFAR2-mediated inhibition of RIG-I/NF-κB signaling, attenuating inflammation and retinal atrophy [72]. Dietary modulation offers a complementary strategy. Omega-3 supplementation increased butyrate-producing bacteria and reduced retinal MHC class II expression, indicating dampened ocular immune activation [73].

Evidence from fecal microbiota transplantation further supports the pathogenic potential of dysbiosis. Transplantation of feces from patients with active neuromyelitis optica spectrum disorder induced Th17 expansion and elevated pro-inflammatory cytokines in recipient mice [74], providing a rationale for precision microbiome editing approaches integrating metabolic and immunomodulatory interventions [75]. Together, these findings position microbial restoration as a foundational strategy within the virus-microbiome synergy model of glaucoma. Precision microbiome editing approaches integrating metabolic and immune profiling remain conceptual and await clinical feasibility studies.

### 4.3. Modulation of Autophagy and Proteostasis for Cellular Clearance in Glaucoma

Impaired cellular clearance contributes to TM dysfunction and neurodegeneration in glaucoma. Autophagy inducers such as rapamycin and everolimus ameliorated TGF-β2-induced TM fibrosis by promoting lysosomal degradation of extracellular matrix components [76], while rapamycin also enhanced mitophagy and reduced oxidative stress-induced apoptosis [77]. However, autophagy modulation requires precision. In familial childhood glaucoma associated with DDX58 mutations, excessive interferon signaling and dysregulated autophagy reduced cell viability, a phenotype alleviated by chloroquine rather than autophagy activation [78]. These contrasting findings underscore that both insufficient and excessive autophagy may be pathogenic depending on disease context.

Alternative strategies include mTOR-independent modulation. Trehalose enhanced aqueous humor outflow by upregulating autophagy-related genes and suppressing thrombospondin-1 expression [79]. Parallel to autophagy, proteasomal regulation is critical for proteostasis. Citicoline acts as a bimodal allosteric modulator of the 20S proteasome, enhancing proteolytic activity and supporting neuronal protein turnover [80]. These clearance-based interventions may be particularly relevant in virus-associated glaucoma, where persistent infection and microbiome-driven immune dysregulation impose sustained proteostatic stress.

### 4.4. Neuroprotection and Immunomodulation Targeting Cellular Senescence and Inflammation

IOP reduction alone often fails to halt glaucomatous neurodegeneration, underscoring the need for neuroprotective strategies. In the context of the virus-microbiome axis, this is particularly critical because chronic infectious stress and sustained pro-inflammatory signaling impose a persistent metabolic and inflammatory burden that accelerates cellular senescence in ocular tissues. RGC exposed to such chronic inflammatory stress acquire senescence-associated features, including DNA damage and nuclear envelope defects [81]. Pharmacological targeting of this process has shown promise; roflumilast attenuated microglial senescence and suppressed NLRP3 inflammasome activation, preserving retinal structure in ischemia-reperfusion models [82].

Redox and epigenetic pathways further modulate neuroinflammation. Melatonin prevented RGC degeneration by activating NRF2 and Sirt1 signaling [83], while the Sirt1 activator SRT2104 reduced neuroinflammation through deacetylation of NF-κB and STAT3 [84]. Natural compounds such as quercetin also exhibited anti-inflammatory and neurotrophic effects via inhibition of TLR4/NF-κB signaling [85]. These approaches offer dual benefits: they counteract neurodegeneration and mitigate immune exhaustion driven by chronic viral and dysbiotic stress. As these approaches remain largely preclinical, they must be interpreted cautiously, given that long-term safety and efficacy in human glaucoma have not yet been established.

### 4.5. Advanced Drug Delivery Systems and Regenerative Therapies for Glaucoma Management

Ocular anatomical barriers, particularly the BRB, critically constrain the translation of therapies targeting the virus–microbiome axis. Conventional topical antivirals or immunomodulators often fail to achieve sustained exposure in the posterior segment where latent viruses and neuroinflammation reside [86]. Accordingly, advanced delivery systems originally developed for IOP control must be repurposed to ensure precise delivery of these novel agents. Nanoparticle-based formulations have improved drug retention and efficacy, including memantine-loaded PLGA-PEG nanoparticles that reduced RGC loss and bimatoprost-loaded solid lipid nanoparticles that sustained hypotensive effects [87,88]. These carriers offer a viable template for encapsulating hydrophobic antiviral agents or unstable microbial metabolites. Injectable hydrogels, such as those containing nano-brinzolamide [89], and implantable systems like intracameral bimatoprost implants or brimonidine-loaded silicone implants [90,91], address adherence challenges. Unlike daily drops which cause concentration fluctuations, these sustained-release platforms are essential for maintaining the constant antiviral exposure necessary to prevent viral reactivation in glaucoma.

Non-invasive platforms such as nanoparticle-laden contact lenses have also improved ocular bioavailability [92]. Finally, regenerative strategies leveraging extracellular vesicles from mesenchymal stem cells or retinal progenitor cells have shown neuroprotective effects by inhibiting RGC apoptosis [93,94]. These platforms are particularly advantageous for virus and microbiome-associated glaucoma, as they can bypass ocular barriers to deliver high concentrations of therapeutic cargo directly to the site of chronic low-grade inflammation (Table 1).

## 5. Challenges and Future Directions

The synergistic interplay between viral infection, microbial dysbiosis, and neuroinflammation offers a novel framework for glaucoma management, redefining the disease beyond a mere neuropathy of elevated IOP. However, bridging the chasm between these mechanistic insights and tangible clinical benefits requires overcoming substantial scientific and translational barriers. The transition from identifying mere associations in the microbiome to establishing causality remains the primary hurdle, demanding a rigorous shift in experimental design. Furthermore, the deployment of systemic interventions targeting the gut–eye axis or cellular autophagy introduces complex safety considerations, necessitating a delicate balance between therapeutic efficacy and the preservation of host homeostatic resilience. Consequently, the field stands at a critical juncture where future progress will depend not on discovering new targets, but on validating causality, developing precision diagnostic tools, and engineering humanized models that faithfully recapitulate this complex dual-hit pathogenesis. The following subsections critically examine these bottlenecks and propose strategic directions for the next generation of glaucoma research.

### 5.1. Validating Causal Mechanisms in Virus-Microbiome Pathogenesis

While the hypothesis of synergistic disease development offers a compelling framework, translating this concept into clinical practice faces substantial hurdles. A primary challenge lies in deciphering causality from association. The majority of current studies linking the gut microbiome to glaucoma are cross-sectional, establishing correlations with specific taxa, including *Butyrivibrio* and *Coprococcus*. However, they fail to definitively prove directional causation [70]. As noted in neurodegenerative research, despite encouraging results linking dysbiosis to disease, many questions remain regarding the specific roles of fungi or viruses, necessitating a transition from descriptive profiling to mechanistic validation. Future research must leverage longitudinal cohort studies and gnotobiotic animal models to unequivocally demonstrate that dysbiosis is a driver, rather than a consequence, of neurodegeneration [95].

### 5.2. Developing Multi-Omics Biomarkers for Precision Diagnosis

Despite the promise of multi-omics, significant technical challenges remain. Integrating viral and bacterial datasets is complicated by batch effects, lack of standardized sampling protocols, and the immense heterogeneity of the human microbiome. Furthermore, distinguishing transient viral signatures from the background genetic noise of the host requires rigorous bioinformatic filtering, which is currently inconsistent across studies.

The heterogeneity of patient responses necessitates the development of precision diagnostic tools. Given that alterations in the gut microbiota can lead to pathological changes and alter metabolite formation, these microbial signatures hold promise as novel biomarkers for disease onset and progression [96]. The field must advance towards utilizing multi-omics integration to identify specific microbial markers that predict therapeutic response [75]. Future research should focus on integrating single-cell multi-omics with dynamic functional assessments to detect the specific signatures of gut–eye axis dysregulation before irreversible damage occurs [97].

### 5.3. Optimizing Safety and Specificity of Emerging Therapies

The safety and specificity of emerging therapies remain a critical bottleneck. While novel targets like TFEB show promise for retinal diseases, it is imperative to obtain well-controlled direct experimental evidence to confirm efficacy while minimizing the risk of adverse effects [98]. For instance, non-specific autophagy activation might disrupt cellular homeostasis if not tightly regulated. Furthermore, the BRB continues to impede the effective delivery of novel agents. Effective restoration requires multi-faceted approaches, and future strategies must balance anti-inflammatory efficacy with barrier-strengthening mechanisms to prevent self-perpetuating cycles of neuroinflammation [97].

### 5.4. Recapitulating the Human Ocular Microenvironment

Finally, bridging the translational gap requires the adoption of more physiologically relevant experimental models. Current rodent models often fail to fully recapitulate the complex chronic inflammatory landscape of the human eye. Emerging technologies such as organ-on-a-chip models and biomaterial-based drug delivery systems are now being highlighted as essential tools for personalized medicine [97]. These platforms will allow for the high-throughput screening of synergistic therapies in a humanized context, ensuring that adaptive therapeutic strategies can be developed to prevent irreversible vision loss.

## 6. Conclusions

Our understanding of glaucoma pathogenesis is shifting from being solely a pressure-dependent neuropathy to a multifaceted systemic disorder involving the “host-virus-microbiome” triad. This review synthesizes emerging evidence suggesting that viral persistence and microbial dysbiosis may disrupt ocular immune privilege and autophagic homeostasis. However, this “dual-hit” model, where gut dysbiosis primes the immune response and viral infection triggers neuroinflammation, remains conceptual and requires further validation.

Current evidence supporting the convergence of viral and microbial factors is primarily derived from experimental models and associative studies, with limited direct causal data. Although these overlapping pathways provide a compelling rationale for therapeutic strategies targeting immune-metabolic imbalance, more rigorous human studies are needed to confirm their role in glaucoma progression.

The clinical management of glaucoma must evolve beyond simply lowering eye pressure. Precision diagnostics that combine viral surveillance and microbiome profiling, along with advanced immunomodulation delivery systems, could enable personalized, etiology-based neuroprotection to preserve vision.

**Figure 4:**
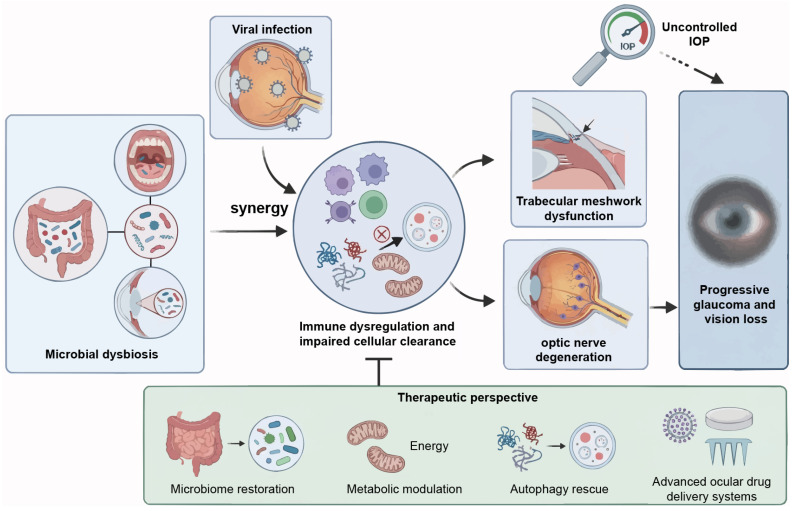
The “host-virus-microbiome” triad and etiology-based therapeutic evolution. The schematic summarizes the paradigm shift from pressure-dependent neuropathy to a multifaceted systemic disorder. Synergistic pathogenesis: Viral persistence and microbial dysbiosis exert a synergistic “dual-hit” effect. This interaction disrupts ocular immune privilege and autophagic homeostasis, driving immune dysregulation and impaired cellular clearance. Clinical progression: These convergent pathways precipitate TM dysfunction and optic nerve degeneration. This mechanism elucidates progressive vision loss despite conventional IOP control. Therapeutic perspective: Clinical management evolves toward precision neuroprotection. Strategies integrate microbiome restoration, metabolic modulation to restore bioenergetics, and autophagy rescue. Advanced ocular drug delivery systems ensure targeted intervention to preserve vision. TM, trabecular meshwork; IOP, intraocular pressure.

Future research integrating virome, microbiome, and host immune signatures will be crucial to confirm whether these pathways cooperate in human glaucoma. This will guide more effective, individualized treatment strategies (Figure 4).

## Figures and Tables

**Table 1:** Integrated Therapeutic Strategies Targeting the Virus–Microbiome–Glaucoma Axis.

Therapeutic Domain	Specific Targets and Interventions	Molecular Mechanism of Action	Translational Outcome and Key Evidence	Evidence Level/Model Source
Infection Control	Antivirals (e.g., Acyclovir, Valganciclovir) Antibiotics (e.g., Azithromycin)	Inhibits viral DNA polymerase and replication; Clears atypical bacterial co-factors (e.g., *Spiroplasma*).	Resolution of secondary IOP elevation driven by HSV/VZV keratouveitis [64]. Pre-surgical inflammation control in infection-associated congenital glaucoma [66].	Clinical glaucoma and ocular disease-specific evidence
Gut–Eye Axis	Metabolic Modulation (e.g., butyrate, phenylbutyrate) Dietary Intervention (e.g., Omega-3 PUFAs)	FFAR2 Activation: Suppresses RIG-I/NF-κB signaling to block viral replication. Systemic Regulation: Lowers IOP via blood-pressure-independent pathways.	Antiviral protection in TM cells against ZIKV [72]. Reduced retinal neuroinflammation via downregulation of MHC-II expression [73].	Extrapolated from CNS/systemic and retinal inflammation models (no direct glaucoma-specific clinical data)
Cellular Clearance	Autophagy Inducers (e.g., rapamycin, trehalose) Proteasome Modulators (e.g., citicoline)	Lysosomal Degradation: Clears fibrotic ECM (collagen I) and damaged mitochondria. Proteostasis: Allosteric activation of the 20S proteasome.	Amelioration of TM fibrosis (TGF-β2-induced) and prevention of oxidative apoptosis [76,77]. Enhanced aqueous outflow via suppression of thrombospondin-1 [79].	Ocular preclinical models (trabecular meshwork and retinal cell/animal studies)
Neuroprotection	Senolytics (e.g., roflumilast) Epigenetic Modulators (e.g., melatonin, SRT2104)	Senescence Inhibition: Targets aging microglia and NLRP3 inflammasome. Redox Regulation: Upregulates NRF2/Sirt1; deacetylates NF-κB.	Preservation of retinal structure by reversing cellular senescence [81,82]. Stabilized visual fields and reduced RGC degeneration [83,84].	Retinal neurodegeneration and ischemia preclinical models (not glaucoma-specific clinical data)
Precision Delivery	Nanoparticles (e.g., PLGA-PEG, SLNs) Exosomes (e.g., MSC-derived EVs)	Barrier Penetration: Enhances posterior segment delivery. Regenerative Signaling: Delivers miR-22-3p to modulate MAPK pathways.	Sustained IOP reduction (up to 21 days) with injectable hydrogels [89]. Significant neuroprotection via cell-free regenerative therapy [93,94]	Ocular drug delivery and regenerative preclinical models (animal and in vitro)

Evidence levels were classified as follows: Clinical glaucoma and ocular disease-specific evidence indicates data derived from human glaucoma or infection-associated ocular disease cohorts; Ocular preclinical models refer to animal or in vitro models involving trabecular meshwork, retina, or optic nerve injury; Extrapolated evidence denotes findings derived from CNS or systemic inflammatory/metabolic models without direct glaucoma-specific validation. Abbreviations: IOP, intraocular pressure; HSV, herpes simplex virus; VZV, varicella-zoster virus; ZIKV, Zika virus; TM, trabecular meshwork; PUFAs, polyunsaturated fatty acids; FFAR2, free fatty acid receptor 2; RIG-I, retinoic acid-inducible gene I; NF-κB, nuclear factor kappa B; MHC-II, major histocompatibility complex class II; ECM, extracellular matrix; TGF-β2, transforming growth factor beta 2; NRF2, nuclear factor erythroid 2-related factor 2; Sirt1, sirtuin 1; NLRP3, NOD-like receptor family pyrin domain-containing 3; PLGA-PEG, poly(lactic-co-glycolic acid)-polyethylene glycol; SLNs, solid lipid nanoparticles; MSC, mesenchymal stem cell; EVs, extracellular vesicles; miR-22-3p, microRNA-22-3p; MAPK, mitogen-activated protein kinase.

## Data Availability

The study did not generate or analyze any new data.

## Abbreviations

The following abbreviations are used in this manuscript:

APP/PS1	amyloid precursor protein/presenilin 1
BRB	blood-retinal barrier
cGAS	cyclic GMP-AMP synthase
CMV	cytomegalovirus
ECM	extracellular matrix
EPA	eicosapentaenoic acid
EVs	extracellular vesicles
FFAR2	free fatty acid receptor 2
HIV	human immunodeficiency virus
*H. pylori*	*Helicobacter pylori*
HSV	herpes simplex virus
IFN-α,	interferon alpha
IFN-β	interferon beta
IL-6	interleukin 6
IL-18	interleukin 18
IOP	intraocular pressure
MAPK	mitogen-activated protein kinase
MHC-II	major histocompatibility complex class II
miR-22-3p	microRNA-22-3p
MSC	mesenchymal stem cell
mtDNA	mitochondrial DNA
NLRP3	NOD-like receptor family pyrin domain-containing 3
NF-κB	nuclear factor kappa B
NRF2	nuclear factor erythroid 2-related factor 2
PLGA-PEG	Poly (lactic-co-glycolic acid)-polyethylene glycol
POAG	primary open-angle glaucoma
PUFAs	polyunsaturated fatty acids
RGC	retinal ganglion cell
RIG-I	retinoic acid-inducible gene I
SCFAs	short-chain fatty acids
Sirt1	sirtuin 1
SLNs	solid lipid nanoparticles
STING	stimulator of interferon genes
STX17	syntaxin 17
TFEB	transcription factor EB
TGF-β1	transforming growth factor beta 1
TGF-β2	transforming growth factor beta 2
TLR	Toll-like receptor
TLR4	Toll-like receptor 4
TM	trabecular meshwork
TNF-α	tumor necrosis factor alpha
VPS39	vacuolar protein sorting 39
VZV	varicella-zoster virus
ZIKV	Zika virus
